# The Heart of Transformation: Exploring Artificial Intelligence in Cardiovascular Disease

**DOI:** 10.3390/biomedicines13020427

**Published:** 2025-02-10

**Authors:** Mohammed A. Chowdhury, Rodrigue Rizk, Conroy Chiu, Jing J. Zhang, Jamie L. Scholl, Taylor J. Bosch, Arun Singh, Lee A. Baugh, Jeffrey S. McGough, KC Santosh, William C.W. Chen

**Affiliations:** 1 Division of Basic Biomedical Sciences, Sanford School of Medicine, University of South Dakota, Vermillion, SD 57069, USA; mohammed.chowdhury@coyotes.usd.edu (M.A.C.); conroyyh@gmail.com (C.C.); jing.zhang@coyotes.usd.edu (J.J.Z.); jamie.scholl@usd.edu (J.L.S.); arun.singh@usd.edu (A.S.); lee.baugh@usd.edu (L.A.B.); 2 Health Sciences Ph.D. Program, Department of Public Health & Health Sciences, School of Health Sciences, University of South Dakota, Vermillion, SD 57069, USA; 3 Pulmonary Vascular Disease Program, Brigham and Women’s Hospital, Boston, MA 02115, USA; 4 AI Research Lab, Department of Computer Science, University of South Dakota, Vermillion, SD 57069, USA; rodrigue.rizk@usd.edu; 5 Department of Psychology, University of South Dakota, Vermillion, SD 57069, USA; taylor.bosch@usd.edu; 6 Department of Electrical Engineering and Computer Science, South Dakota School of Mines and Technology, Rapid City, SD 57701, USA

**Keywords:** artificial intelligence, machine learning, cardiovascular disease, disease diagnosis, disease prediction

## Abstract

The application of artificial intelligence (AI) and machine learning (ML) in medicine and healthcare has been extensively explored across various areas. AI and ML can revolutionize cardiovascular disease management by significantly enhancing diagnostic accuracy, disease prediction, workflow optimization, and resource utilization. This review summarizes current advancements in AI and ML concerning cardiovascular disease, including their clinical investigation and use in primary cardiac imaging techniques, common cardiovascular disease categories, clinical research, patient care, and outcome prediction. We analyze and discuss commonly used AI and ML models, algorithms, and methodologies, highlighting their roles in improving clinical outcomes while addressing current limitations and future clinical applications. Furthermore, this review emphasizes the transformative potential of AI and ML in cardiovascular practice by improving clinical decision making, reducing human error, enhancing patient monitoring and support, and creating more efficient healthcare workflows for complex cardiovascular conditions.

## 1. Introduction

Cardiovascular disease is the leading cause of mortality worldwide, responsible for 17.9 million deaths annually that represent 32% of all global deaths, and continues to be a conundrum scientists and clinicians have been trying to solve for decades [1]. It is predicted that by 2030, the number of cardiovascular-related deaths could reach over 20 million every year due to the increasing aging populations all over the world [2,3]. Furthermore, the associated economic burden is expected to rise substantially by 2050, from USD ~400 billion to USD ~1500 billion annually [4].

There are many types of highly debilitating and lethal cardiovascular diseases, such as cardiac arrhythmia, coronary artery disease, peripheral arterial disease, and heart failure (HF), impacting one’s quality of life substantially. Many risk factors lead to cardiovascular disease, including genetic background, obesity, diabetes, smoking, and underlying or pre-existing disease conditions such as hypertension and coronavirus disease 2019 (COVID-19). In particular, COVID-19 infections, which have surged in recent years, may result in myocarditis, pericarditis, and thromboembolism [5,6]. Therefore, the capacity to integrate the patient’s information from all sources, including family history, lifestyle, medical records, physical examination, blood tests, imaging studies, biopsies, genetic investigation, and other clinical and bioinformatic data, will be the key to improving disease diagnosis, intervention planning, and even patient prognosis. Artificial intelligence (AI) and machine learning (ML) offer a solution by eliminating the burden of interpreting sophisticated tables and performing tedious calculations.

**Figure 1:**
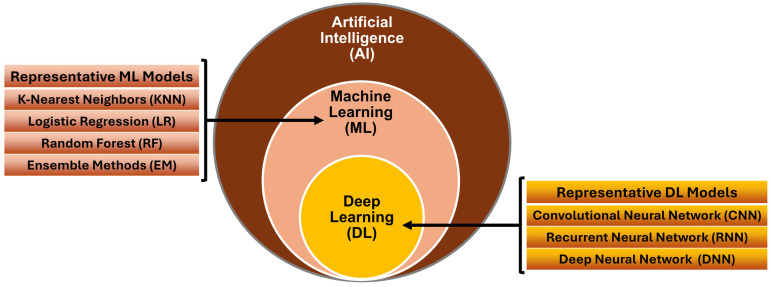
Relationships between artificial intelligence (AI), machine learning (ML), and deep learning (DL). Commonly used ML and DL models are listed, respectively.

In recent years, the integration of AI and ML has catalyzed transformative advancements across various domains of biomedicine [7]. AI and ML techniques are revolutionizing the healthcare landscape by offering powerful tools to (1) analyze complex biological and biomedical data, (2) improve diagnostic accuracy, (3) personalize treatment regimens, and (4) optimize healthcare delivery [8,9,10,11,12]. AI encompasses a spectrum of methodologies that enable machines to simulate human cognitive functions such as learning, reasoning, and problem solving (Figure 1). ML, a subset of AI, focuses on algorithms that automatically learn patterns and make predictions from data without explicit programming (Figure 1). Together, AI and ML have demonstrated significant capabilities in biomedical applications, ranging from medical imaging analysis [13] and genomic sequencing [14] to drug discovery [15] and clinical decision support systems [16].

Furthermore, rapid advances in deep learning (DL), a branch of ML, have significantly enhanced the efficiency of interpreting complex signals and patterns in medicine (e.g., medical images) and enabled more precise detection and characterization of diseases such as cancer and neurological disorders (Figure 1) [13]. Natural language processing (NLP) techniques, utilized extensively in AI/ML tools, have streamlined the analysis of unstructured clinical big data, extracting valuable insights to support clinical decision making and biomedical research [17]. In particular, predictive modeling powered by AI/ML can revolutionize healthcare and public health systems by predicting disease trajectories, identifying at-risk populations, and optimizing therapeutic strategies based on precision medicine—tailored to one’s genetic background, family history, and medical record [18]. These approaches have demonstrated substantial promise in improving patient classification, diagnosis, treatment selection, and prognosis and facilitated the development of personalized therapy based on individual patient profiles [14,19].

These advancements in AI and ML have also significantly impacted cardiovascular medicine, enhancing diagnostic and treatment decisions. AI/ML applications are being developed to diagnose, manage, and predict a wide variety of heart conditions, such as coronary artery disease (CAD), arrhythmias, and HF [20,21]. ML models are being integrated into clinical workflows to predict patient outcomes, determine disease trajectory and prognosis, and identify high-risk patients for adverse events [22,23]. Moreover, with the availability of DL models, digital biomarkers such as wearable monitoring devices are being utilized with other diagnostic modalities to aid clinicians’ decision making and to develop personalized management plans for each patient [17,24]. These advancements have been driven by collaborations between medical professionals and data scientists, leveraging large datasets and powerful computational models to transform clinical cardiovascular care and improve patients’ prognoses [19,25]. The contributions of this systemic literature review include the following:

(1) Summarizing the recent progress of AI/ML applications in cardiovascular disease, particularly between 2022 and 2024.
(2) Exploring commonly used AI/ML models and approaches in many different frontiers of cardiovascular care and health management.
(3) Discussing the challenges, limitations, and potential solutions of AI/ML applications in cardiovascular disease.
(4) Introducing policy and ethical considerations of AI/ML applications in cardiovascular disease.
(5) Highlighting the promise of AI/ML applications and their future utility.

## 2. The Utility of AI in Cardiovascular Disease

AI/ML has emerged as a transformative tool in cardiovascular medicine, revolutionizing how we approach risk stratification, disease diagnosis, treatment selection, prognostication, and prediction, ultimately improving patient outcomes [26,27]. AI/ML systems can analyze vast amounts of patient data with unparalleled accuracy through user-defined algorithms and models, enabling early detection of cardiovascular diseases, precise risk assessment, and personalized treatment strategies.

**Figure 2:**
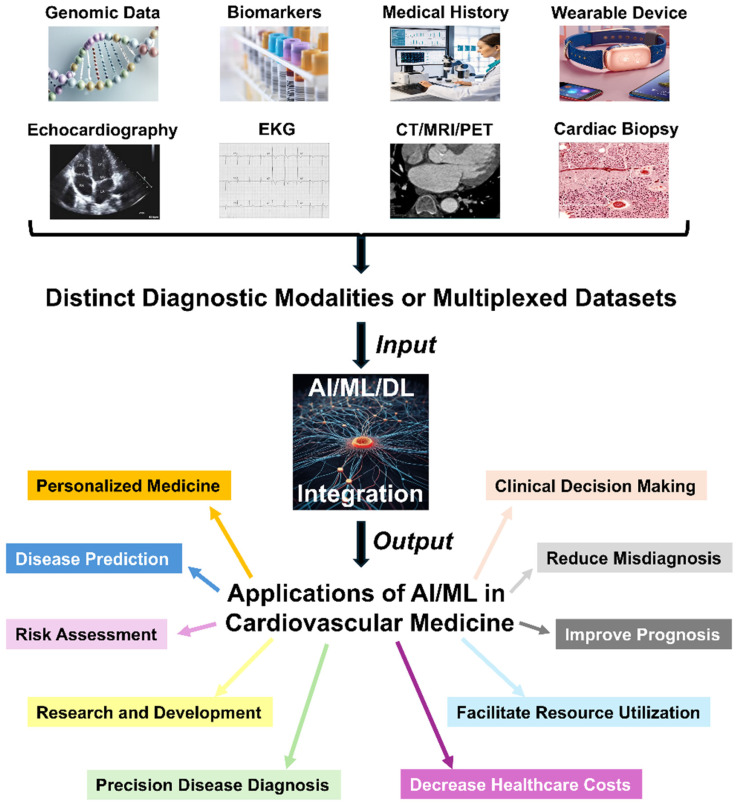
Summary of current and investigative applications of AI/ML in cardiovascular medicine.

These technologies have been demonstrated to be instrumental in detecting, diagnosing, and treating cardiovascular conditions, offering new opportunities for improving patient outcomes and advancing medical research [28]. In this section, we introduce how AI and ML can be applied in different aspects of cardiovascular disease management (Figure 2).

### 2.1. Disease Diagnosis

Cardiovascular disease can often be silent, especially at early stages, and thus remains challenging for clinicians to diagnose. Misdiagnosis is a major cause of delayed treatment, which not only wastes precious diagnostic time and resources but may eventually harm the prognosis of patients. In a review of 3407 malpractice cases from general medicine clinics, cardiovascular cases were more likely to involve allegations of diagnostic errors than non-cardiovascular cases (75% vs. 47%, *p* < 0.0001) [29]. In medical physics and imaging, especially in radiology, AI/ML systems have played increasingly crucial roles in assisting clinicians with image analysis, disease diagnosis, radiology decision support systems, and radiotherapy treatment planning [13,30]. Moreover, the development of AI-based algorithms for image classification in neurological and psychiatric diseases showcases the potential of AI/ML to improve clinical diagnosis and efficiency [31]. As a result, there has been a growing interest in using AI/ML as an assistive tool to integrate patient data from multiple diagnostic modalities with distinct clinical features and help recognize subtle patterns of disease presentation not evident to most clinicians to improve diagnostic accuracy and aid clinical decision making. AI/ML models are being actively developed for several commonly used cardiac diagnostic modalities.

#### 2.1.1. AI Utility in Electrocardiograms (EKGs)

Hannun et al. developed a deep neural network (DNN) algorithm using 91,232 single-lead EKGs [32]. They could classify 12 heart arrhythmias more accurately than board-certified cardiologists (F-measure 0.84 vs. 0.78) [32]. Moreover, convolutional neural networks (CNNs) developed with the input of substantial numbers of digital EKGs linked to clinical datasets were reported to be capable of analyzing EKGs and diagnosing asymptomatic left ventricular dysfunction, silent atrial fibrillation (AF), and hypertrophic cardiomyopathy (HCM), even predicting the individual’s age, sex, and race [33]. DL models that pair EKG data with information on right and left ventricular function from echocardiogram reports extracted by NLP could classify left ventricular function, estimate left ventricular ejection fraction with a reported mean absolute error of only 5.84%, and predict a composite outcome of right ventricular systolic dysfunction or dilation with an area under the curve (AUC) (i.e., probability) of 0.84 [34]. Therefore, EKG data analyzed by AI models can be used as a potential screening tool to diagnose cardiac diseases.

#### 2.1.2. AI Utility in Echocardiograms

Echocardiograms play a significant role in cardiology, ranging from screening for heart disease to the characterization and diagnosis of cardiomyopathies. AI applications in echocardiography can be an efficient solution to reduce diagnostic errors because AI/ML models trained with sufficient amounts of existing clinical echocardiogram data can perform (1) recognition of the standard cardiac sections, (2) automatic segmentation of the cardiac cavity, (3) functional assessment of the left ventricle, and (4) cardiac disease diagnosis [35]. For example, Zhang et al. reported that a CNN model developed with 14,035 echocardiogram studies was able to (1) accurately identify 23 viewpoints and segmentation of cardiac chambers across the five standard views and consequently identify the different cardiac chambers based on that, (2) accurately measure cardiac volumes, and (3) discriminate diseases from healthy controls [36]. Similarly, an ML algorithm based on clinical and echocardiographic parameters could differentiate HCM from the athlete’s physiologically hypertrophic heart with a sensitivity of 87% and specificity of 82% [37].

#### 2.1.3. AI Utility in Cardiac CT and MRI

The utility of AI/ML as a diagnostic tool has been widely explored in radiological imaging modalities such as cardiac computed tomography (CT), positron emission tomography (PET), and magnetic resonance imaging (MRI). Cardiac CT is commonly used to calculate the calcium score for patients at increased risk of CAD and is an established method to determine a patient’s long-term prognosis [38]. An AI model using a CNN was reported to accurately evaluate cardiac CT exams and classify calcium scores with an accuracy rate of 83% [39]. Wang et al. used artificial neural networks (ANNs) to improve PET imaging of myocardial perfusion, achieving significant image quality enhancement and noise reduction without compromising diagnostic accuracy [40]. Moreover, the ANN fusion technique significantly improved the detectability of the non-transmural and transmural defects in the myocardium [40]. Kwiecinski et al. employed ML to combine 18F-Sodium Fluoride PET imaging with quantitative plaque analysis on CT angiography, demonstrating an AUC of 0.85 for predicting future myocardial infarction (MI) [41]. ML algorithms using non-enhanced cine cardiac MRI detected chronic MI with 90% sensitivity and 99% specificity [42]. These studies suggest that AI/ML has the potential to automate the interpretation of cardiac imaging modalities with high sensitivity and specificity and thus help reduce human error, aid in clinical decision making, and improve the use of resources, especially in medical centers where there is a shortage of personnel with expertise in image processing and interpretation.

#### 2.1.4. AI Utility in Other Aspects of Diagnostic Imaging

Other utilities of AI/ML in diagnostic imaging include improving workflow efficiency and image quality, reducing the dose of radiation or contrast agents, and analyzing human behavior patterns to identify patients at higher risk of missing their appointments. In one study, telephone reminders sent to high-risk patients identified by these AI/ML models reduced the no-show rate from 19.3% to 15.9% [43]. Additionally, the diagnostic accuracy of AI/ML in imaging studies has been improving, leading to reduced reading time and workload for radiologists [44,45]. A generator CNN, trained to convert low-dose CT images into routine-dose CT images with voxel-wise loss minimization, allowed estimation of coronary calcium scores from low-dose CT scans with high noise levels [46]. This study suggests that AI/ML models could potentially assist radiologists in obtaining quantifiable images with lower contrast agents or radiation doses than conventional imaging techniques. Furthermore, AI/ML has exhibited significant potential in acquiring and post-processing radiological images [47].

#### 2.1.5. Future AI Utility for Cardiovascular Disease Diagnosis

Currently, the applications of AI and ML technologies are being advanced with the integration of large-scale patient databases, medical expert inputs, and prospective validation processes in the abovementioned diagnostic modalities. For example, by analyzing high-dimensional cardiovascular data from various sources such as EKGs, echocardiograms, and other imaging modalities, AI has enabled the early detection of cardiovascular disease and the assessment of future risks [48]. The FDA approval of AI/ML-enabled medical devices underscores the growing integration of these computational breakthroughs into healthcare systems [49]. In the future, AI/ML can not only enhance clinical decision making but also pave a new way for implementing preemptive population health management and early intervention in public health and clinical practice, respectively, ultimately reducing the prevalence of cardiovascular disease and improving the prognosis and quality of life of the patients. As AI/ML continues to evolve, its integration into cardiovascular medicine promises to redefine the standards of modern medicine, ushering in a new era of precision medicine and proactive healthcare management.

### 2.2. Disease Prediction

AI/ML prediction models are expected to play an important role in modern cardiology by leveraging data-driven algorithms and large-scale clinical datasets to estimate the likelihood of cardiovascular events or diseases in individuals [50]. These models integrate diverse patient variables such as medical history, disease biomarkers, imaging results, and lifestyle factors to generate personalized risk assessments [51,52]. By identifying high-risk individuals early, prediction models enable healthcare providers to implement preventive measures or personalize interventions, potentially reducing morbidity and mortality rates.

AI has tremendous potential in this field, where it can integrate data from multimodal sources to generate reliable, efficient disease predictive models. For example, an ML model that integrated blood cardiac troponin levels with clinical features, including age, sex, comorbidities, and the time between troponin measurements, was used to develop the CoDE-ACS score, which can be used to determine an individual’s probability of developing MI [53]. It has also been reported to have comparable prediction performance to guideline-recommended pathways for MI [54]. ML use in cardiovascular medicine has improved diagnostic accuracy and prognosis and helped identify individuals at high risk of developing cardiovascular diseases [55]. AI has also been integrated with biomechanical modeling to predict cardiovascular disease based on risk factors and medical imaging findings and to assess hemodynamics and vascular geometries indirectly [56].

Despite a steady increase in the development of clinical predictive models for various cardiovascular diseases, their real-world applications are limited. One major challenge to be solved is their poor statistical performance, particularly in terms of their discrimination and calibration when these models are applied to new or diverse populations. Additionally, there is often inconsistency in effect sizes and risk estimates across different predictive models, further limiting their reliability. These limitations reduce their impact on clinical decision making, as clinicians may lack confidence in their accuracy and generalizability [57].

To improve the utility of predictive models in clinics, it is vital to overcome these hurdles by integrating all available patient information to augment their predictive performance. For example, the patient electronic health record (EHR) is a rich source of data and an asset for designing DL algorithms. Mallya et al. reported the development of a Long Short-Term Memory (LSTM) algorithm, a type of recurrent neural network (RNN), using time-series data from over 23,000 patients; the LSTM successfully predicted the onset of HF 15 months before the onset of apparent symptoms with an AUC of 0.91 [58].

Diagnostic imaging modalities are vital to cardiovascular workup and are increasingly used to develop predictive AI/ML models. For example, cardiac CT parameters from 10,030 patients with CAD were used to train an ML model that not only successfully predicted 5-year all-cause mortality but also achieved a higher AUC than commonly used prediction tools, such as the Framingham Risk Score, CCTA severity scores, and the modified Duke Index, suggesting that AI/ML-based predictive models can outperform traditional statistical methods [59]. In cardiovascular imaging, AI has been increasingly used to automate disease detection, enhance diagnostic accuracy and efficiency, assist clinical decision making, guide treatment decisions, improve patient care in cardiovascular emergencies, predict cardiovascular risks and disease outcomes, and identify new drug targets [60,61,62].

### 2.3. Federated Learning for Cardiovascular Disease

Federated learning (FL) is a decentralized ML approach that enables multiple healthcare institutions or organizations (e.g., hospitals, research centers, and medical facilities) to collaboratively train a model without sharing the raw data in a way that would not otherwise be possible due to data-sharing restrictions [63,64]. Different healthcare providers may have access to diverse patient populations from distinct geographic, racial, ethnic, and socioeconomic backgrounds, which could provide more comprehensive datasets for training. This helps in improving the diagnosis process, developing accurate predictive models, and improving healthcare outcomes, where patient data privacy and confidentiality are critical concerns [64,65,66,67].

Cardiovascular disease data are highly sensitive, such as medical history, diagnostic images (e.g., X-ray and CT and MRI scans), EKG results, blood tests, and genetic data. Additionally, cardiovascular disease (e.g., heart attacks, strokes, hypertension, and arrhythmias) are often influenced by a wide range of factors, including age, gender, lifestyle, genetics, and comorbid conditions. Different healthcare institutions may have access to varying types of medical data (e.g., patient demographics, imaging, genomics, EHRs, etc.). FL enables institutions to retain control over their data without sharing the data directly while collaboratively contributing to training a shared model, leading to a more accurate and robust model that can handle the complexity of CVD diagnosis and prediction. The risk of exposing personal health information is minimized by processing the data locally and only sharing model updates (i.e., gradients or parameters) [68,69], in contrast to the traditional methods that rely on centralizing data for training [65,70].

However, the deployment of FL in healthcare is not without challenges. Issues such as data heterogeneity, communication efficiency, and model convergence need to be addressed to fully realize the potential of FL [71,72,73]. Additionally, ensuring the security of model updates against potential attacks, such as data poisoning, remains a critical concern [74]. Overall, FL represents a promising privacy-preserving approach for advancing AI-based cardiovascular disease management and research and may play an essential role in the future of healthcare informatics.

## 3. Current AI Models for Cardiovascular Disease

**Figure 3:**
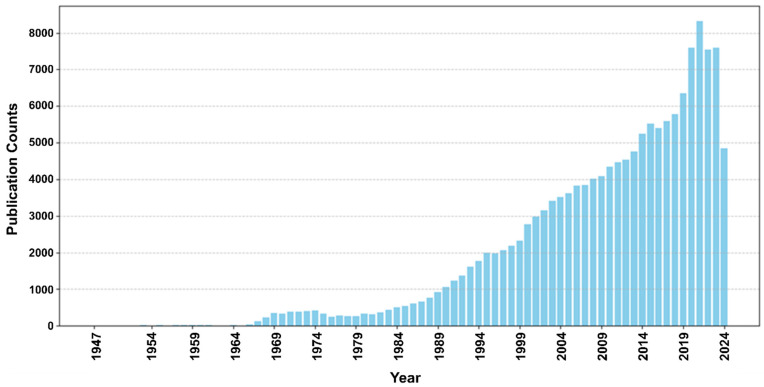
Cumulative AI publications related to cardiovascular disease. This graph shows the cumulative numbers of AI papers on cardiovascular disease in PubMed from 1947 to 2024.

AI models have recently emerged as helpful clinical tools, revolutionizing how cardiovascular diseases are diagnosed, managed, and predicted. AI/ML algorithms can integrate and analyze complex multimodal datasets, including medical images, genetic information, and clinical records, to detect subtle patterns and correlations within immense amounts of seemingly unrelated data. Since the emergence of AI, its growth in cardiovascular science has been surging, especially in the past 5 years, reflected by the significantly increasing numbers of publications related to AI and ML in cardiovascular disease (Figure 3). AI-based technologies hold great promise for cardiovascular disease management, such as real-time detection of arrhythmias through wearable devices, non-invasive diagnosis of pathological conditions (e.g., CAD), efficient planning of personalized treatment for severe cases (e.g., HF), and accurate prediction of patient outcomes [27]. Currently, there are several AI approaches available for cardiovascular applications, and the choice of an ML technique depends on several factors: the particular healthcare application, the needed information and solution for the specific clinical question, and the type of data (structured versus unstructured) available to train the model (Table 1). In this section, we introduce common ML and DL models that have been tested or utilized in cardiovascular medicine and discuss relevant studies and use cases.

### 3.1. Machine Learning Models

(1) K-nearest neighbors (KNN): KNN is a simple yet effective algorithm that classifies data points based on their proximity to other data points. KNN has been used to identify similar patients based on clinical features such as age, cholesterol level, and blood pressure [82].
(2) Logistic regression (LR): LR is widely used for binary classification tasks. It has been used to estimate the probability of an individual developing cardiovascular disease based on risk factors. It is interpretable and valuable for understanding the impact of different features [82].
(3) Random forest (RF): RFs combine multiple decision trees to improve predictive accuracy. They handle complex interactions between features and are robust against overfitting. RFs perform well due to their ensemble nature [82].

### 3.2. Deep Learning Models

(1) Convolutional neural network (CNN): CNNs excel at processing image data. For cardiovascular medicine, CNNs can perform specific tasks to aid clinical diagnosis and treatment planning, such as segmenting and classifying heart images [82].
(2) Recurrent neural network (RNN): RNNs are useful for time-series data, such as monitoring patients’ vital signs over time. RNNs can be used to predict disease progression or to detect anomalies [82].
(3) Deep neural network (DNN): With their multiple hidden layers, DNNs can learn complex representations from diverse patient data. DNNs are valuable for risk prediction and personalized treatment recommendations [82].
(4) Ensemble methods (EMs): EMs combine multiple ML/DL models to enhance performance. For example, XGBoost, a gradient-boosting algorithm and a widely used EM approach, has been successful in various medical applications, including cardiovascular disease prediction [83].

### 3.3. Other Models and Use Cases

Srinivasan et al. compared eight published ML techniques for predicting cardiovascular disease and reported that neural network models, including Naïve Bayes and Radial Basis Functions, achieved prediction accuracies of 94.78% and 90.78%, respectively, for heart disease predictions, while in comparison, Learning Vector Quantization demonstrated the overall best performance, with an accuracy rate of 98.7% [84]. In a meta-analysis of 344 studies evaluating the predictive performance of commonly used ML algorithms for cardiovascular disease, the authors reported that for the prediction of CAD, custom-build algorithms (AUC 0.93) performed better than boosting algorithms (AUC 0.88) [79]. For the prediction of stroke, several ML predictive models had similar performance: support vector machine (SVM) algorithms, boosting algorithms, and CNN algorithms had pooled AUCs of 0.92 (95% CI 0.81–0.97), 0.91 (95% CI 0.81–0.96), and 0.90 (95% CI 0.83–0.95), respectively [79]. However, for HF and cardiac arrhythmias, while insufficient studies were found for meaningful meta-analysis, SVM appeared to have performed better than other models [79].

Another study reviewing the MI national registry consisting of 755,402 patients reported that ML models did not substantially improve the prediction of in-hospital mortality compared to LR [85]. However, the extreme gradient descent boosting and meta-classifier algorithms (C-statistic 0.9) were better classifiers than LR (C-statistic 0.89) and improved calibration algorithms across the CAD risk spectrum, accurately determining an individual’s risk for adverse outcomes [85]. Therefore, the search and debate for the best ML approach for cardiovascular disease applications continues, as demonstrated by the abovementioned studies. However, as AI advances, with a better understanding of this technology by clinicians and biomedical programmers, the predictive performance of these models may improve over time.

Another concern with ML prediction models is the lack of capacity to capture the sequence or temporality of events [86]. Typical ML approaches, such as LR or RF algorithms, cannot adequately capture temporal information. As a result, these methods represent patient events of interest only as an aggregated count or as a summary measure, lacking temporality of events [54]. RNNs, on the other hand, can encode time-stamped events from EHR data and learn latent representations for use in classification tasks [86]. Thus, the RNN may be a better model than conventional ML approaches when temporal information is available in the datasets and could be a contributing factor. For example, to predict the future diagnosis of HF, Choi et al. utilized an RNN (gated recurrent units [GRUs]) to detect temporal relations among time-stamped events (diagnostic history, medication orders, procedure orders, etc.) in EHRs [87]. By applying GRUs to the time-stamped events, they could capture and model the progression and dependencies of these events across time. Briefly, data from EHRs of 3884 incident HF cases and 28,903 controls were analyzed over a 12-to-18-month observation period, and the performance of the RNN model in predicting new diagnosis of HF was compared with conventional ML approaches that cannot capture temporality, including regularized LR, neural networks, support vector machines, and K-nearest neighbor classifiers [87]. The RNN model performed better than all temporality-irrelevant ML approaches in the 12-month (AUC 0.77) and 18-month windows (AUC 0.88) [87]. The GRU structure was particularly beneficial in this application because it used gating mechanisms to selectively update the network’s hidden state at each time step. Thus, it learned to keep or ignore inputs (diagnoses, medications, procedures) as it saw fit. Therefore, to develop a comprehensive ML model for clinical applications, we may need to ensure that the learning algorithm includes all events of interest across a designated time rather than consolidating them as a single, aggregated event.

## 4. AI in Cardiovascular Disease Diagnosis, Management, and Prognostication

AI and ML are increasingly transforming cardiovascular disease management, particularly in disease prediction, prognostication, risk assessment, and personalized medicine. By learning from a sufficient number of datasets from clinical records, imaging, genomics, and other data, AI/ML algorithms can identify subtle patterns of correlation that lay the foundation to fulfill the predictions of the onset, progression, and outcome of cardiovascular disease, possibly enabling early, tailored treatment plans for individual patients. These technologies also enhance risk assessment by accurately stratifying patients based on their likelihood of developing pathological conditions like cardiac arrhythmias, MI, HF, or cardiogenic shock, allowing for more precise interventions. Thus, through AI and ML, personalized medicine can be further advanced by customizing therapies based on comprehensive individual patient profiles, ultimately improving and optimizing the outcomes of the patients with better utilization of limited healthcare resources.

### 4.1. Cardiovascular Research

AI/ML has been increasingly integrated into cardiovascular research to augment and accelerate scientific discovery. AI/ML can help scientists identify knowledge gaps, design experiments, collect and interpret large datasets, and gain insights that might not have been possible using conventional scientific methods [88]. For instance, AI can recover missing data in EHRs and improve the quality of data available for retrospective studies [89]. NLP, a component of AI allowing computers to understand human text and speech, has been reported to extract patient diagnosis and symptoms from medical charts with accuracies of 91% and 90.6%, respectively [90]. An NLP model developed using a multi-institutional EHR from the Mass General Brigham healthcare system exhibited the capacity to successfully recover missing clinical data, including vital signs and other essential information, which may significantly reduce bias in studies and enhance the generalizability of research findings [91]. Moreover, in a multicenter clinical trial, the N model was shown to identify HF events, and its performance accurately approximated human reproducibility [90]. Similarly, an ML-based data processing pipeline has improved missing data imputation in an ICU database for patients with acute coronary syndrome, significantly enhancing the performance of the cardiogenic shock prediction model trained on this database [89].

### 4.2. Myocardial Infarction (MI)

MI has estimated annual incidences of 605,000 new attacks and 200,000 recurrent attacks in the United States alone [3]. MI is caused by acute occlusion of coronary arteries and often results in considerable myocardial damage, ischemic cardiomyopathy, and fatal arrhythmias that may lead to HF or cardiac arrest in many patients. Therefore, AI/ML models that can predict adverse outcomes and recurrence following MI may help identify patients at risk from HF or death and guide timely and appropriate interventions. For example, an ANN with a backpropagation algorithm developed using 21 variables from the SWEDEHEART registry consisting of 139,288 patients was able to predict the majority of all-cause mortality and HF-associated admission in patients who suffered from MI [92]. For 1-year all-cause mortality, the ANN had an AUC of 0.85 (95% CI 0.84–0.85) in the testing dataset and 0.84 (95% CI 0.83–0.84) in the external validation cohort. For HF-associated admission to a hospital within 1 year of the MI insult, the ANN had an AUC of 0.82 (95% CI 0.81–0.82) in the testing dataset and 0.78 (95% CI 0.77–0.79) in the external validation dataset [92].

### 4.3. Cardiac Arrhythmia

Ventricular tachycardia (VT) and ventricular fibrillation (VF) are abnormally fast, life-threatening heart rhythms commonly seen in patients with MI and ischemic cardiomyopathy [93]. The MI-induced scar formation in the cardiac tissue, along with a meandering network of surviving cardiomyocytes interspersed within the fibrosis, results in discontinuous and anisotropic slow conductions that lead to the development of re-entrant VT or VF development [94,95]. Arevalo et al. developed a Virtual-heart Arrhythmia Risk Prediction (VARP) approach that non-invasively assessed the risk of sudden cardiac death from ventricular arrhythmia using cardiac MRI and computational modeling [96]. With the VARP, they could recreate a personalized three-dimensional heart model to study each patient with MI. The VARP heart model incorporated individual patients’ ventricular geometry, MI-associated structural remodeling, and electrical functions from the sub-cellular to the organ levels to predict one’s risk of developing sudden cardiac death and the need for implantable cardioverter defibrillators (ICDs) after MI; this model significantly outperformed present clinical metrics in predicting future arrhythmic events [96].

Currently, catheter-based radiofrequency ablation is the standard treatment of VT/VF; however, it uses electrical mapping to identify target areas that are often inaccurate, resulting in a modest success rate of 50–88% [97]. The VARP approach was reported to identify targets and guide VT ablation more accurately when compared with the current approach utilizing conventional electrical mapping [98].

Similarly, AI/ML gradually establishes its role in atrial fibrillation (AFib), a highly prevalent cardiac rhythm abnormality affecting 46.3 million people globally [99]. AFib ablation by catheterization is the current standard of care for patients with symptomatic AFib. However, the commonly utilized clinical scores to predict the long-term success of AFib ablation have suboptimal discrimination, with an AUC ranging between 0.55 and 0.65 [100]. A CNN model based on intracardiac signals, EKGs, and patient clinical features notably improved the prediction accuracy of successful AFib ablation by catheterization (AUC 0.86), compared with models based on clinical scores that did not incorporate intracardiac signals [100]. Moreover, AI-guided catheter ablation demonstrated superior performance to the present catheter ablation in terms of acute AFib termination rate (78% vs. 10%, *p* < 0.001) and freedom from AFib at 12 months (89% vs. 67%, *p* = 0.001) [101].

### 4.4. Heart Failure (HF)

The cardiopulmonary exercise test (CPET) is the standard diagnostic modality for the long-term prognosis of HF patients and the determination of their needs for cardiac transplantation or a left ventricular assist device (LVAD) [102]. Current clinical strategies for patient prognostication are mainly based on limited summative indices that may not accurately estimate the disease trajectory. In contrast, ML can acquire and integrate multiple input indices from different time points, notably improving the performance of disease prediction models [103]. A feedback neural network (FNN) model trained with CPET time-series data allowed the incorporation of temporal data from discrete stages within the test and each breath-by-breath cycle. Compared with other conventional multivariable prediction models for clinical deterioration in HF patients (e.g., needing mechanical circulatory support, listing for heart transplantation, or mortality from any cause), the neural network model integrating breath-by-breath data exhibited the best performance (AUC 0.84) [103]. Similarly, Adler et al. developed an ML model by training a boosted decision tree algorithm using a cohort of 5822 hospitalized and ambulatory patients with HF [104]. This model generated a mortality prediction score with superior performance (AUC 0.88) compared to the clinical mortality scores [104].

### 4.5. Right Ventricular Failure (RVF)

RVF is associated with increased morbidity and mortality, with a two-year mortality of 45% compared with 7% in those without RVF [105]. RVF is commonly seen in patients with right ventricular MI and those who have increased right ventricular afterload due to pulmonary hypertension, cardiac surgery, or an LVAD [105]. Due to the complex anatomy of the right ventricle, preoperative assessment of right ventricular dysfunction using echocardiography remains challenging [106]. This challenge remains because it is difficult to obtain complete visualization of the entire right ventricle, and echogenicity may be suboptimal in certain patients [107]. An AI analytical system that can process videos from echocardiograms was developed to simultaneously input and analyze two parallel spatiotemporal streams of data, including the grayscale video channel and the optical flow channels, and then combine the results within the CNN architecture, followed by concatenation of activations to produce an RVF prediction model [108]. For post-operative RVF, the echocardiographic video-trained AI system outperformed not only the commonly used predictive score but also a team of human experts at the same task on independent evaluation, with an AUC of 0.729 [108].

### 4.6. Cardiogenic Shock (CS)

CS is the leading cause of death post-MI, complicating 5–10% of all MI cases [109]. Over the last two decades, the 6–12 month mortality rate has remained very high at 50% in CS patients [110]. To improve clinical decision making and intervention efficacy, several AIML-based models have been developed to aid cardiologists and recognize impending CS and adverse outcomes [111,112,113]. For example, an ML consensus clustering analysis (CCA) was shown to synthesize clinical and laboratory data patterns to reveal distinct CS phenotypes with different clinical outcomes [111]. Similarly, an algorithm based on LR could identify patients with elevated risk for developing CS [112]. Furthermore, an ML model based on the XGBoost algorithm successfully predicted the onset of CS and aided clinical decision making with an AUC of 0.87 [113].

### 4.7. Mechanical Circulatory Support (MCS)

MCS is commonly used to stabilize patients with refractory CS. Extracorporeal membrane oxygenation (ECMO), a widely used form of MSC, provides life-saving support to circulatory and pulmonary systems. A DNN-trained ECMO predictive algorithm based on the Extracorporeal Life Support Organization (ELSO) registry predicted in-hospital mortality better than the current scoring systems, with a sensitivity of 82.1 ± 0.2% and precision of 77.6 ± 0.2% [114]. Another study demonstrated that an ML model could accurately predict survival to hospital discharge in patients receiving veno-arterial extracorporeal membrane oxygenation (VA-ECMO) [115]. By analyzing clinical variables and patient data, the ML model identified patterns associated with survival, offering a potential tool for improving clinical decision making and patient outcomes in this high-risk population.

LVADs are durable mechanical circulatory pumps used in patients with end-stage HF as a destination therapy or as a bridge to cardiac transplantation. AI-based applications are being investigated to improve the management of patients with LVADs, including prediction of post-LVAD survival [116,117,118], RVF based on echocardiogram parameters and hemodynamic data [119], aortic insufficiency [120], LVAD-related adverse outcomes [117], and myocardial recovery in LVAD-implanted patients [121]. Driveline infections are frequent complications in patients with LVADs and pose significant challenges to patient care, as they can lead to serious complications that affect both device functions and patient outcomes [122]. A recent study reported that an ML model can review the pictures of the driveline insertion sites uploaded by patients to a remote server and classify the sites as being clean or infected with an accuracy of 93.75%, sensitivity of 100%, and specificity of 87.5% [123].

### 4.8. Cardiac Transplantation

Cardiac transplantation is one of the most complex medical conditions to manage, requiring frequent short- and long-term monitoring for rejection, side effects of immunosuppression, and life-threatening complications such as cancer [124]. However, current monitoring approaches often rely on invasive procedures, making them limited in predictive accuracy and relatively incapable of detecting subtle signs of early rejection or adverse effects. Integrating AI/ML may help predict patient-specific risks, provide non-invasive tools for monitoring patients, and enhance early detection of unwanted events to reduce complications and improve outcomes. The use of AI in this field is increasingly being investigated with various ML approaches that can predict organ waitlist mortality [22], post-transplantation survival [125,126,127,128], and graft rejection [129]. The International Heart Transplantation Survival Algorithm (IHTSA) is a flexible, nonlinear ANN model that can comprehensively evaluate the impact of recipient–donor variables on patient survival over time [130]. The IHTSA model predicted both short- and long-term post-transplant mortality with high accuracy (C-index 0.600 [95% CI: 0.595–0.604]); IHTSA also had a superior discrimination power (0.650 [95% CI: 0.640–0.655]) compared with the current risk models of post-transplant mortality such as the donor risk index (DRI) (0.56 [95% CI: 0.56–0.57]), the risk-stratification score (RSS) (0.61 [95% CI: 0.60–0.61]), and the index for mortality prediction after cardiac transplantation (IMPACT) (0.61 [0.61–0.62]) [130]. Moreover, the added capabilities of DL in IHTSA for capturing nonlinear and hidden patterns resulted in error reductions by 12% and 10% in the prediction of short-term and long-term mortality when compared with the traditional models, respectively [131].

Patients with cardiac transplantation undergo routine endomyocardial biopsies to screen for histological evidence of tissue rejection. EKGs paired with endomyocardial biopsies were used to develop a DL model that detects allograft rejection from the EKG [132]. The AI-EKG detected acute cellular rejection with an AUC of 0.84 and 95% sensitivity, suggesting that it can be used as an alternative modality for tissue rejection screening and possibly replace endomyocardial biopsies [132]. In another study, AI was used to automate the process of tissue rejection surveillance where endomyocardial biopsy slides were used to train attention-based DNNs to detect cellular patterns of rejection (ISHLT grade [0R, 1R, 2/3R]) and predict post-transplant rejection, resulting in a satisfactory prediction performance with an AUC of 0.849 [133]. Moreover, ML models have been reported to guide the administration of immunosuppression by estimating the blood levels of immunosuppressive drugs Cyclosporine and Tacrolimus in heart transplant recipients [134,135].

### 4.9. Inherited and Rare Cardiovascular Diseases

Recent advances in human genetics and sequencing technologies rapidly improve our understanding of a variety of inherited and rare cardiovascular diseases, including cardiomyopathies (e.g., dilated cardiomyopathy, HCM, and arrhythmogenic cardiomyopathy), arrhythmic disorders (e.g., long QT syndrome, short QT syndrome, and Brugada syndrome), vascular disorders (e.g., Ehlers–Danlos syndrome and Marfan syndrome), and lipid disorders (e.g., familial hypercholesterolemia) [136]. However, diagnosing genetic cardiovascular disorders remains challenging because it requires extensive manual curation and interpretation of candidate genetic variants, which is a highly labor-intensive task even for trained geneticists [137]. To reduce the burden on geneticists, Mao, Liu, and Wang et al. developed AI-MARRVEL (AIM), an ML-based automatic variant prioritization tool with phenotype context that employs a random-forest ML classifier trained on over 3.5 million genetic variants from thousands of diagnosed cases of Mendelian diseases [137]. AIM was reported to improve the diagnosis of genetic disorders with a high precision rate of 98%, suggesting the promise of applying mature AI/ML models in this clinical field [137].

### 4.10. Pulmonary Hypertension (PH)

Pulmonary hypertension (PH), characterized by elevated pulmonary pressures, is a rare disease with a prevalence between 5 and 15 cases per 1 million adults [138]. It is often challenging to diagnose patients with PH mainly because patients present with a gradual onset of shortness of breath and require a high index of suspicion and exclusion of other cardiopulmonary pathologies. AI is rapidly proving its utility in this specialty [139]. For example, an ML approach based on clinical symptoms has been developed to identify patients at elevated risk for developing PH six months before the onset of the disease (AUC 0.84, sensitivity 0.73, and precision 0.5), using an RF algorithm that retrospectively analyzed US healthcare claims data to establish the model [140]. Another ML model based on clinical and echocardiographic parameters, such as right ventricular systolic pressure, size, and function, was able to successfully diagnose PH with accuracy, sensitivity, and positive- and negative-predictive values of 82%, 88%, 89%, and 54%, respectively [141]. Moreover, an RF-based model was reported to identify patients who developed PH due to concomitant left heart disease with a sensitivity of 70% and a specificity of 100% [142].

### 4.11. Cardiac Amyloidosis (CA)

Cardiac amyloidosis, a debilitating disease with poor life expectancy, is an infiltrative cardiomyopathy caused by the systemic deposition of abnormal proteins produced by plasma cells that misfold and deposit in cardiac tissues and other organs, leading to organ damage and dysfunction [143]. CA is often difficult to diagnose because its symptoms mimic common heart conditions, and its early signs can be subtle or nonspecific, leading to frequent misdiagnosis or delayed recognition by clinicians. Two recent studies reported successful applications of AI-enhanced EKGs for early detection of CA, suggesting the possibility of timely diagnosis and intervention for patients with CA before the onset of clinical cardiac dysfunction [144,145].

### 4.12. Cardio-Oncology

The field of cardio-oncology focuses on the prevention and management of cardiovascular consequences of cancer therapies. The current strategy for the surveillance of anti-cancer chemotherapy-related cardiotoxicity includes regular monitoring through imaging techniques (e.g., echocardiography) and cardiac biomarkers (e.g., troponins and BNP/NT-proBNP) that detect early signs of heart damage. However, the present clinical surveillance protocol has inherent limitations in identifying and predicting individuals with high risks of developing drug-related cardiomyopathy [146]. AI/ML-based models are currently being explored to monitor cardiotoxicity and improve patient outcomes in this specialty. One such application is the use of CNNs to analyze the EKGs of patients receiving anti-cancer chemotherapy and then computationally predict the onset of cardiac dysfunction or atrial fibrillation [146].

### 4.13. Implantable and Wearable Medical Devices

AI/ML is setting a new milestone for big data-based cardiology research and patient care by learning and integrating numerous real-time data from implantable and wearable medical devices. Multiple randomized controlled trials have supported the feasibility of AI in analyzing and interpreting continuous physiological data captured by wearables, such as smartwatches and EKG monitors [147,148,149]. For example, in a study evaluating the efficacy of smartwatches equipped with an irregular pulse notification algorithm to screen for new onset of AFib, the authors reported that 34% of the participants who received a notification had AF on subsequent EKG readings, with a positive-predictive value of 0.84 (95% CI, 0.76 to 0.92) [150]. These modern innovations, along with data-driven insight and proactive healthcare management, would empower early detection of cardiovascular abnormalities, facilitate remote monitoring, and enable personalized diagnostic and treatment strategies, enhancing therapeutic outcomes and patient prognosis in modern cardiology.

### 4.14. Improving Healthcare Resource Utilization

There is an ongoing shortage of healthcare personnel in the United States. The Association of American Medical Colleges (AAMC) predicted that by 2030, the demand for doctors will outstrip the supply and that the United States is expected to experience a shortage of up to 121,300 physicians, especially in rural areas [151]. Moreover, in the post-COVID-19 pandemic era, there has been an increasing shortage of nurses due to unsafe work environments or occupational burnout [152]. In a 2021 national survey by the American Association of Critical-Care Nurses, about 66% of all respondents reported considering leaving the profession [153]. The shortage of nurses will significantly impact the healthcare sector since increased nurse staffing is associated with lower odds of hospital-related mortality and adverse patient events [154].

In addition, with the advancement in medical technology, growing numbers of intricate drugs and therapies, and increased sophistication in clinical care, healthcare costs will continue to rise. The US spending on cardiovascular disease and management of cardiovascular risk factors increased by more than USD 100 billion (about USD 310 more per person in the US) from 1996 (USD 212 billion) to 2016 (USD 320 billion) [155]. AI/ML has the potential to tremendously affect this sector by improving the efficiency of healthcare resource allocation and utilization, reducing the incidents of diagnostic errors and unnecessary medical procedures, and expanding health insurance and healthcare administrative costs. Integrating AI/ML models into hospital patient monitoring systems and in intensive care units has increased the accuracy of alarms for adverse events, improving patient outcomes, reducing unnecessary procedures, facilitating resource allocation, and decreasing healthcare personnel fatigue due to false alarms [156,157,158,159].

Moreover, several AI/ML-based applications are being developed to improve ambulatory patient monitoring, for example, using a chatbot computer program to check up on patients after hospital discharge, monitor patient health at home, and respond to patient messages and queries [160]. The application of mature AI/ML-based technologies in healthcare is expected to reduce the workload of physicians, nurses, and ancillary staff and increase the efficiency and appropriateness of resource allocation and utilization in healthcare systems. Furthermore, AI/ML applications are being actively investigated in patient health management for the outpatient monitoring of medication adherence and drug-related toxicities and side effects [161,162]. For example, AI/ML was used to monitor current health conditions and symptoms in patients with chronic diseases and to alert their healthcare providers when necessary to prevent complications and reduce hospital readmissions [163].

### 4.15. Reducing Healthcare Disparities

Online and remote access to AI-based tools or AI-assisted medical equipment holds great promise for addressing healthcare disparities in resource-constrained and rural environments. Cloud-based platforms and integrative telemedicine enable the deployment of AI-driven diagnostic or decision support systems in regions and populations with limited access to medical specialists or advanced healthcare infrastructure [164]. These AI-based tools can remotely analyze patient data, rapidly offer diagnostic suggestions, and efficiently assist in triaging cases to ensure timely interventions. Additionally, mobile applications and lightweight AI algorithms optimized for low-bandwidth settings can further extend the reach of these tools to underserved regions [165,166]. However, the effectiveness of these potential solutions for healthcare disparities is often limited by inadequate hardware, unreliable internet connectivity or signal transmission, and/or insufficient infrastructure in many underpopulated areas. These challenges must be addressed to ensure the seamless implementation and sustainability of AI technologies in such settings.

### 4.16. Knowledge Gaps Between Modeled and Real Clinical Practice

Despite the growing exploration of AI applications in healthcare, its adoption in clinical settings has been limited. Zhou et al. reviewed 65 randomized clinical trials evaluating AI-based clinical interventions and found that nearly 40% of these studies show no clinical benefits from using AI prediction tools, compared to the standard care [167]. Although the AI models demonstrated strong performance metrics during development and validation, they did not outperform traditional risk-stratification calculators [167]. This conclusion highlights a notable gap between the performance of AI models trained and tested with defined datasets and their performance in real patient populations, which must be addressed before a successful integration of AI tools into routine clinical practice.

## 5. Challenges of AI in Cardiovascular Disease

Although AI has the potential to revolutionize cardiovascular disease care and management, there are several caveats and challenges that we need to be aware of or overcome before AI can be accepted as a standard healthcare practice.

(1) A significant concern with AI/ML applications in clinics is the integrity of the patient data used to train the AI/ML algorithms. This concern is inevitable because an AI/ML model can only be as good as the data used to train this ML model. There is heterogeneity in the quality of distinct cardiovascular clinical studies, including how they were performed, interpreted, and stored within different institutions and practices. Moreover, the incompleteness of data samples, noise in the data, and the stochastic nature of the modeling algorithm can significantly affect the accuracy of AI/ML models [168].

(2) Other major challenges for AI/ML applications in healthcare include proper data sourcing, curating, sharing, and privacy protection [162]. Sourcing high-quality, representative data is critical but often hampered by fragmented EHRs and inconsistent data standards across institutions [169]. Curating data for ML models also requires significant effort to clean, label, and structure them for meaningful insights, whereas data sharing between healthcare systems is limited due to concerns over patient privacy, regulatory compliance, and data security [169]. Thus, a universal big data platform that reliably integrates appropriately processed healthcare data and safely shares them among institutions without violating patient privacy may be necessary to facilitate clinical AI/ML applications.

(3) AI in biomedicine now extends to privacy-preserving techniques: it ensures the security of patient data while leveraging AI’s potential for medical advancements [170]. Ultimately, robust privacy protection measures, such as de-identification and encryption, are essential to safeguard sensitive patient information while fostering AI-driven advancement for healthcare [171]. For example, when using diagnostic images for AI/ML training, imaging data from clinical repositories could be difficult to obtain, and these data may often be unstructured or unlabeled [162]. This issue is further exaggerated because ML patterns frequently overfit a dataset mainly because of their inability to differentiate true contributing factors from noise (i.e., irrelevant or random data that distort the input), thus leading to inaccurate prediction or reduced model performance. This is one of the main reasons why an AI/ML model may underperform or cannot replicate the prediction accuracy of previous datasets when presented with a new dataset in an external validation study [172].

(4) Another important issue with healthcare-oriented AI/ML is data bias, especially concerning the diverse patient populations in different racial, ethnic, geographic, religious, and socioeconomic groups worldwide. An expert or a clinician must appropriately annotate data on clinical incidents/events before they can be used for AI/ML training. Errors in the annotations, differences in medical expert opinions, and distinct diagnostic thresholds among physicians could introduce biases during the annotation process, consequently affecting the performance of the AI/ML model trained with the biased datasets. Furthermore, the majority of cardiovascular clinical trials have underrepresented women and minority groups, and using datasets derived from those trials may lead to biased learning and skewed performance [173]. New studies focusing on the underrepresented patient groups may be needed to tackle the insufficient data availability.

(5) There is a lack of standardized external, prospective validation strategies to fairly evaluate the long-term efficacy of the trained AI/ML models [174]. As a result, the accuracy and the generalizability of the AI/ML models, particularly those unvalidated ones, in real-life patients should still be interpreted cautiously. A comprehensive review of 486 AI prediction models and screening tools for cardiovascular disease revealed that none has undergone independent external validation, and most are at high risk of bias, with only 10 deemed “recommended” for clinical use [25]. This is another reason why a degree of skepticism remains among clinicians and patients when adopting AI/ML-based applications in healthcare. To help improve the utility of AI prediction models in cardiovascular domains, the editors for digital health, innovation, and quality standards of the European Heart Journal proposed five minimal quality criteria for AI-based prediction model development and validation studies: (a) complete reporting, (b) carefully defined intended use of the model, (c) rigorous validation, (d) large-enough sample size, and (e) openness of the code and software [175]. Recently, there has been increased emphasis on developing tools to assess the methodological and reporting quality of AI prediction models designed for clinical decision support [176].

(6) Physicians often face challenges in understanding and interpreting the complex decision-making processes of ML and DL models, hindering their utilization in daily clinical practice. To address this critical issue, explainable AI techniques have been developed, such as Shapley Additive Explanations (SHAP) and Local Interpretable Model-Agnostic Explanations (LIME) [177,178]. These methods aim to improve model transparency by elucidating how the models function, thereby increasing the end-user trust in their outputs [179].

To achieve the accuracy obtained in the validation cohort in clinical practice, the AI/ML models must undergo rigorous external validation [180]. While each AI/ML approach has its own set of strengths and weaknesses, it is imperative that an appropriate model is carefully chosen for the intended task based on the nature of data available for training and the needs of the clinical problem to be addressed. The utility of AI/ML in healthcare should improve with time as our experience with this new technology increases and physicians and programmers better understand the full potential of AI/ML-based applications.

## 6. Policy and Ethical Considerations of AI in Cardiovascular Disease

Regulatory policy and ethical concerns surrounding the use of AI and ML in cardiovascular disease and other fields are critical areas of focus in healthcare that directly impact the public acceptance of these new technologies. Integrating AI/ML technologies in cardiovascular care has shown promise in improving early disease detection, individualized treatment regimens, and patient outcomes [26,181]. Despite the potential benefits, significant ethical considerations need to be addressed to ensure the responsible and trustworthy use of these technologies in healthcare settings [182,183,184].

Studies have highlighted the importance of transparency, trust, and ethical considerations from the perspectives of patients, caregivers, and healthcare providers when utilizing AI/ML in cardiovascular care [182]. Although AI algorithms demonstrate high sensitivities, specificities, and accuracies in detecting heart disease, ethical concerns must be addressed while implementing these models in clinical practice [183]. Furthermore, the gap between AI research and clinical practice in cardiovascular science underscores clinicians’ need to be knowledgeable about AI-based technologies and approaches to ensure the safe and effective integration of these valuable tools into their clinical practice [185].

To resolve the contradiction between privacy protection and data sharing in AI applications for cardiovascular disease, several strategies can be employed, including easy-to-understand and transparent user consent mechanisms, patient data anonymization or pseudonymization, minimal data collection of only absolutely necessary information, adoption of FL and differential privacy, strong access controls and security measures, and compliance with data privacy regulations such as the Health Insurance Portability and Accountability Act (HIPAA) in the United States [186,187,188]. Implementing some or all of those strategies would be critical to ensure that clinical datasets are safely used only for the intended purposes by the AI models while protecting individual patient identities and abiding by the regulations.

Ethical guidelines and regulatory policies are vital in shaping healthy research, development, and deployment of AI/ML technologies in healthcare [189,190]. Integrating ethics into AI development requires a systemic and comprehensive perspective that focuses on healthcare applications and encompasses ethical considerations from distinct cultural, religious, racial, socioeconomic, and other societal backgrounds [190]. Importantly, the involvement of AI in the clinical decision-making process and the need for explainable and transparent AI for public policymaking require the highest ethical standards and rely on political attention and legislative effort to responsibly ensure compliance and accountability for the general public’s best interests [191,192].

## 7. The Future of AI in Cardiovascular Disease

The future of AI and ML in clinical medicine holds great promise for revolutionizing how cardiovascular disease is diagnosed, managed, and treated. For example, AI/ML/DL algorithms have been investigated to directly diagnose cardiovascular diseases, including HCM, HF, mitral regurgitation, aortic stenosis, arterial and pulmonary hypertension, and CAD, showcasing the versatility of these technologies in clinical practice [22,193]. ML algorithms are already being employed in cardiovascular imaging to automate disease detection, and experts anticipate that ML will further transform the field in the coming years [60]. With a significant number of ongoing AI-related investigations and efforts in the field, coupled with parallel progress in big data and precision medicine, widespread utilization of innovative AI tools and approaches in cardiovascular sciences, diagnostics, therapeutics, and personalized medicine appears to be promising in the near future [22,194].

AI-driven tools are expected to reduce human errors and enhance the accuracy and speed of cardiovascular disease detection through improved data integration and predictive analytics [195]. AI/ML/DL algorithms can leverage patient big data in large databases with multidimensional variables to find subtle patterns and overcome the limitations of current risk models, enabling more precise prediction of patient outcomes or early interventions [196]. Additionally, AI can aid in developing personalized treatment plans by integrating informative data from various sources, including genetic background, lifestyle factors, physical examinations, imaging and blood works, and family and medical history, ultimately improving patient prognosis and reducing healthcare costs. As AI evolves and matures, it may soon bring transformative changes to clinical practice and revolutionize cardiovascular medicine.

## Figures and Tables

**Table 1:** Examples of the performance of AI models for cardiovascular disease. This table shows examples of AI models commonly used for cardiovascular disease and their representative performance in the literature.

Study	Data	AI Model	Performance
Dritsas et al. [75]	Clinical	Stacking Ensemble Model with Synthetic Minority Oversampling TEchnique (SMOTE)	87.8% accuracy, 88.3% recall, 88% precision, and 98.2% AUC
Bhat et al. [76]	Kaggle	Multi-Layer Perceptron (MLP)	87.28% accuracy
Bashaar et al. [77]	Clinical	ANN, Gradient-Boosting Machine (GBM), SVM, RF	ANN: OR of 0.0905, CI of [0.0489; 0.1673]; GBM: average accuracy of 91.10%; SVM: OR of 25.0801, CI of [11.4824; 54.7803]; RF: OR of 10.8527, CI [4.7434; 24.8305]
Lee et al. [78]	Wearable Devices	DNN	AUROC of 0.981
Krittanawong et al. [79]		SVM, Boosting Algorithms, CNN	SVM: AUC of 0.92; Boosting Algorithms: AUC of 0.91; CNN: AUC of 0.90
Mohan et al. [80]	Clinical	RF with a Linear Model	88.7% accuracy
Abdar et al. [81]	Clinical	SVM	93.08% accuracy, 91.51% F1-score
